# Male intake of omega-3 fatty acids and risk of intimate partner violence perpetration: a nationwide birth cohort – the Japan Environment and Children's Study

**DOI:** 10.1017/S2045796022000294

**Published:** 2022-06-23

**Authors:** Kenta Matsumura, Kei Hamazaki, Akiko Tsuchida, Hidekuni Inadera, the Japan Environment and Children's Study (JECS) Group

**Affiliations:** 1 Department of Public Health, Faculty of Medicine, University of Toyama, Toyama, Japan; 2 Toyama Regional Center for JECS, University of Toyama, Toyama, Japan; 3 Department of Public Health, Gunma University Graduate School of Medicine, Gunma, Japan

**Keywords:** Epidemiology, nutrition, violence/aggression, women

## Abstract

**Aims:**

Intake of omega-3 polyunsaturated fatty acids (PUFAs) has favourable effects on reducing aggressive and violent behaviours, but its association with perpetration of intimate partner violence (IPV) is not known. We aimed to determine the association between male intake of omega-3 PUFAs and risk of IPV perpetration.

**Methods:**

Participants were male–female pairs in the Japan Environment and Children's Study, in which first pregnant women and then their partners were enrolled (analytic sample: *n* = 48 065). Male intake of omega-3 PUFAs during the past year (preconception to mid/late pregnancy) was determined using a food frequency questionnaire. IPV (physical violence and emotional abuse) during pregnancy was measured using a self-reported questionnaire completed by pregnant women in mid/late pregnancy. Generalised additive mixed-model analysis was used to examine the non-linear association between energy-adjusted male omega-3 PUFA intake and the adjusted prevalence of male IPV perpetration.

**Results:**

A sharply decreasing regression curve was plotted for physical violence, with prevalence starting at 1.35% at the lowest intake level and decreasing to a minimum value of 0.76% at intake of 2.20 g/day (71.7th percentile). However, prevalence largely remained flat from there onward, with the upper limit of the error range not reaching the initial lower limit of the error range until intake exceeded 5.21 g/day (99.77th percentile). For emotional abuse, on the other hand, there was a distorted U- or V-shaped regression curve that rose slightly after reaching a minimum. The prevalence declined from 17.69% initially, reached a minimum at 12.44% at 2.13 g/day (68.3th percentile), and then rose slightly. The lower limit of the error range reached the minimum upper limit at 4.17 g/day (99.1th percentile), and the upper limit finally reached the maximum lower limit value at 4.56 g/day (99.5th percentile).

**Conclusions:**

In this nationwide birth cohort study, higher male intake of omega-3 PUFAs was associated with lower risk of physical violence and emotional abuse perpetration except for extremely high intake. Our results indicate the potential applicability of omega-3 PUFAs in reducing aggressive and violent behaviours in IPV.

Trial registration: UMIN000030786.

## Introduction

Intimate partner violence (IPV), one of the most common forms of violence against women, includes physical violence, sexual violence, emotional abuse and controlling behaviours (Krug *et al*., 2002). A recent meta-analysis of 140 studies from 81 countries (Devries *et al*., 2013b) revealed a lifetime prevalence of physical and/or sexual IPV in women older than 15 years of 30.0% (95% confidence interval (CI) 27.8–32.2%). A population-based survey conducted in Japan in 2017 showed similar prevalence (31.3%) (Gender Equality Bureau Cabinet Office, 2017). The consequences of IPV are serious and extensive; for example, IPV is a leading cause of homicide death in women worldwide (Stockl *et al*., 2013) and causes injury, chronic pain, sexually transmitted diseases, depression and post-traumatic stress disorder (Campbell, 2002; Devries *et al*., 2013a).

The many identified risk factors for IPV can be divided into individual, relationship, and community and societal factors (Abramsky *et al*., 2011; World Health Organization and Pan American Health Organization, 2012; Fulu *et al*., 2013). However, many factors are related to cultural and social components and it would therefore be difficult to target these factors in any intervention. One scarcely explored and easily targeted factor that may protect against IPV is male intake of the polyunsaturated fatty acid (PUFA) omega-3. Such fatty acids, present in fish oil, include eicosapentaenoic acid (EPA) and docosahexaenoic acid (DHA). Reviews and a meta-analysis have determined that omega-3 intake can effectively decrease aggressive and violent behaviours (Appleton *et al*., 2008; Hamazaki and Hamazaki, 2008; Gajos and Beaver, 2016). In addition, an international study identified an inverse and country-specific correlation between seafood consumption and homicide mortality rates (Hibbeln, 2001). Also, a Japanese study found that DHA and EPA supplementation decreased interpersonal aggression (Hamazaki *et al*., 1996). Our previous study revealed that higher maternal intake of omega-3 PUFA during pregnancy was associated with a lower risk of infant maltreatment (Matsumura *et al*., 2021). However, no studies have examined the relationship of these favourable properties of PUFAs with IPV.

The present study availed of data from the Japan Environment and Children's Study (JECS), an ongoing nationwide birth cohort study, in which first pregnant women and then their partners were enrolled, and explored the association of male intake of omega-3 PUFAs in the past year with the risk of IPV perpetration. Our hypothesis was that male partners with higher omega-3 PUFA intake would be less likely to perpetrate IPV.

## Methods

### Study design and population

**Fig. 1.**
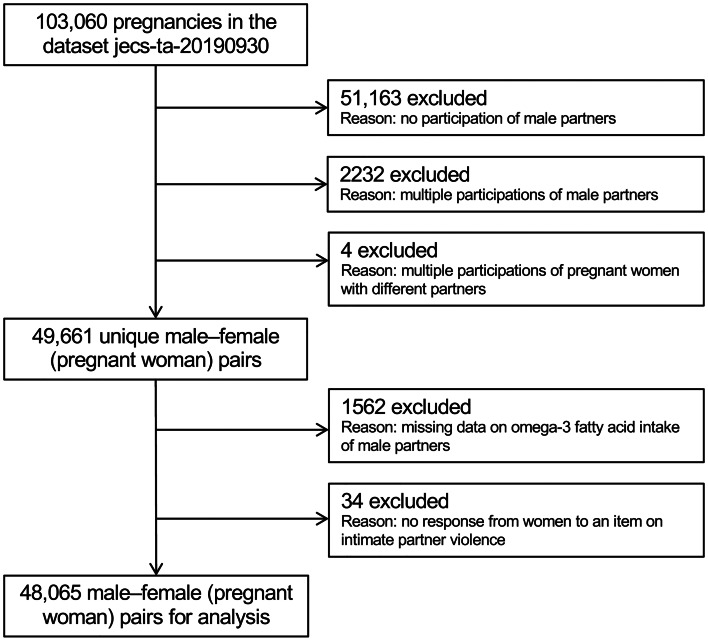
Participant flow chart.

All participants were members of the JECS, an ongoing, nationwide, government-funded birth cohort study whose aim is to explore the effects of environmental factors on child health and development. Its design has been detailed previously (Kawamoto *et al*., 2014; Michikawa *et al*., 2018). Briefly, the study enrolled pregnant women during early pregnancy from 15 regional centres (both rural and urban) in Japan through face-to-face recruitment from January 2011 to March 2014. Follow-up took place in the second or third trimester. Their partners were also recruited when our recruiters were able to meet them, including at delivery. The present study analysed the jecs-ta-20190930 dataset, which was released in October 2019 and contains data on 103 060 pregnancies. To derive unique male–female pairs, we excluded 51 163 pregnant women without participation of the partners, as well as 2232 and four pairs with multiple participations from a second time onward by the male and female partners, respectively. Of the remaining 49 661 pairs, a further 1562 and 34 were excluded due to no response or missing data on male intake of omega-3 PUFAs and the female partner's response about IPV, respectively. In total, 48 065 pairs were ultimately analysed (Fig. 1).

### Measures

A self-administered questionnaire was used to collect information on anthropometric variables, demographic and socioeconomic status, medical and obstetric history, physical and mental health, lifestyle, diet and other topics. The questionnaires were distributed by well-trained JECS staff at cooperating health care facilities twice to pregnant women (during early and mid/late pregnancy) and once to their partners (during pregnancy). Because the questionnaires included highly intimate details, participants were informed that their answers would be anonymised and strictly protected even from their doctors, nurses and partners, and that they would not have to answer any questions they did not want to answer. Some participants completed the questionnaire on the spot at the health care facility, where their privacy was protected. However, most took the questionnaires home, were instructed to complete the questionnaires in an undisturbed environment and returned them on their next visit. Questionnaires were returned by post if they could not be collected.

#### Exposure

Male intake of omega-3 PUFAs during the past year (preconception to present) was measured using a food frequency questionnaire, administered in mid/late pregnancy (mean ± s.d., 27.0 ± 13.0 weeks of gestation). This semi-quantitative, self-administered instrument was developed to evaluate the average consumption of 171 food and beverage items, 21 of which were fish- or shellfish-related, and has been validated for use in large-scale Japanese epidemiological studies (Sasaki *et al*., 2003; Yokoyama *et al*., 2016). With this instrument, participants were asked how often and how much they consumed each food type. Responses were made using nine frequency categories (less than 1 time/month, 1–3 times/month, 1–2 times/week, 3–4 times/week, 5–6 times/week, every day, 2–3 times/day, 4–6 times/day and ⩾7 times/day) and three portion size categories of small (50% smaller than standard), medium (same as standard) or large (50% larger than standard). Intake of each item was determined by multiplying its frequency of consumption by its standardised portion size. Then, omega-3 PUFA intake was determined by summing the intake of each food item multiplied by its omega-3 PUFA content from the fatty acid composition table of Japanese foods (Ministry of Education, Culture, Sports, Science and Technology, 2005). Energy-adjusted omega-3 PUFA intake was calculated using a residual model (Willett *et al*., 1997). The residuals of the linear regression of log-transformed energy intake on log-transformed omega-3 PUFA intake and the predicted log-transformed omega-3 PUFA intake at the mean log-transformed energy intake were added and then back-transformed using an exponential transformation. Because 0 cannot be log-transformed, a log (*x* + 0.5)-type transformation was used to stabilise the variation, where 0.005, 1/2 of the smallest unit of measurement, was used instead of 0.5 as a constant (Yamamura, 1999). Energy-adjusted omega-3 intake was used as a continuous exposure variable.

#### Outcome

IPV perpetration by male partners during pregnancy (learning of pregnancy to present) was assessed by pregnant women via a self-reported questionnaire administered in mid/late pregnancy (mean ± s.d., 27.7 ± 6.3 weeks of gestation). The items regarding IPV were selected and re-organised with reference to a standardised structured questionnaire (Fulu *et al*., 2013), derived from the WHO Multi-country Study on Women's Health and Domestic Violence against Women (Garcia-Moreno *et al*., 2006), while considering its sensitive nature and the highly intercorrelated nature of the items (Fulu *et al*., 2013). The following IPV items were used in the present study:

- *Physical violence*
  - Being hit or beaten up by their husband/romantic partner in the course of a quarrel, which led to injury, during the current pregnancy
- *Emotional abuse*
  - Being insulted or cursed at by their husband/romantic partner, during the current pregnancy

Pregnant women were asked to mark their frequency of experiencing physical violence and emotional abuse on a four-point Likert scale (never, seldom, sometimes and often). In line with the original definition of IPV as any behaviour that causes harm (Krug *et al*., 2002), any answer other than ‘never’ to the IPV items was defined as a case of IPV, which was used as an outcome variable in this study. We note that these two items were embedded as questions 13 and 14 on page 6 of the 27-page questionnaire consisting of 122 main questions (excluding the face sheet). In addition, the partner's questionnaire did not include an item on IPV.

#### Potential confounders

Based on existing evidence and theoretical inference, we selected potential confounders as variables likely to affect both the prevalence of IPV (Krug *et al*., 2002; Abramsky *et al*., 2011; World Health Organization and Pan American Health Organization, 2012; Fulu *et al*., 2013) and the intake amount or physiological (functional) effectiveness of omega-3 (Mozaffarian *et al*., 2005; Itomura *et al*., 2008; Lin *et al*., 2010; Schiepers *et al*., 2010; Thesing *et al*., 2018; de Groot *et al*., 2019). These variables were selected *a priori* and included male age, body mass index, highest education level, annual household income, permanent full-time work, alcohol intake, smoking status, marital status, number of children, history of major psychiatric disease, severe psychological distress and the regional centre they belong to. Some variables (education, income, marital status and number of children) were derived from the pregnant women's answers, but others were derived from the male partners' responses. All variables were categorised according to standard medical practice and common practice in Japan (Matsumura *et al*., 2019). The categorisations are shown in Table 1.

**Table 1.** Characteristics of male partners included in the study

Variable	*n*	(%)
Age, y		
<25	3361	(7.0)
25 to <30	11 312	(23.5)
30 to <35	15 779	(32.8)
⩾35	17 614	(36.6)
Body mass index, kg/m^2^		
<18.5	1760	(3.7)
18.5 to <25	33 184	(69.0)
⩾25	13 121	(27.3)
Highest education level, y		
⩽12	20 296	(42.2)
>12 to <16	11 355	(23.6)
⩾16	16 414	(34.2)
Full-time work		
No	7824	(16.3)
Yes	40 241	(83.7)
Annual household income, million JPY		
<4	18 915	(39.4)
4 to <6	16 143	(33.6)
⩾6	13 007	(27.1)
Smoking status		
Never	13 945	(29.0)
Former	13 625	(28.3)
Current	20 496	(42.6)
Alcohol intake		
Never	10 204	(21.2)
Former	1809	(3.8)
Current	36 052	(75.0)
Number of children		
0	22 335	(46.5)
⩾1	25 730	(53.5)
Marital status		
Married	46 596	(96.9)
Single	1264	(2.6)
Divorced or widowed	205	(0.4)
History of depression, anxiety or schizophrenia		
No	46 528	(96.8)
Yes	1537	(3.2)
Kessler Psychological Distress Scale (K6) score		
0–12	47 124	(98.0)
⩾13	941	(2.0)

Values show the data for 48 065 participants after imputation of missing data.

### Statistical analysis

Characteristics of the male partners were summarised as frequencies. Differences between pregnant women whose partners participated and did not participate were examined using Cramer's *V*, calculated from the *χ*^2^ value. The distribution of energy-adjusted omega-3 intake was summarised using the lower cut-offs of quartiles (i.e. the 0.25th, 25th, 50th, 75th and 99.75th percentiles). Besides the prevalence of IPV, the pattern of IPV was also summarised (i.e. both physical violence and emotional abuse, physical violence only, emotional abuse only or none).

Generalised additive mixed-model analysis, with logit set as a link function and the 15 regional centres as a random effect, was used to examine the non-linear association between energy-adjusted omega-3 PUFA intake and the adjusted prevalence of IPV. The exposure variable was log-transformed energy-adjusted omega-3 PUFA intake with the best-fitting spline as a smoothing function. Outcome variables were cases of each type of IPV. All the potential confounders selected *a priori* were used to adjust the model with the forced entry method. We first obtained their odds ratios (ORs) with their standard error of means (SEMs) and then converted them to adjusted prevalence ± SEM. We plotted a regression curve with energy-adjusted omega-3 PUFA intake on the *x*-axis (range: 0.25th to 99.75th percentile after back-transforming the log-transformed value using an exponential transformation) and each adjusted prevalence of IPV on the *y*-axis.

Missing data were handled by multiple imputation (⩽2% of data for all variables with the exception of 2.74% for number of children and 7.21% for annual household income). Ten imputed datasets were created using chained equations (van Buuren, 2007) and standard rules were used to combine the results (Rubin, 2004). All analyses were performed using SAS version 9.4 software (SAS Institute Inc.) and R 4.0.5.

#### Sensitivity analysis

To assess the robustness of the results, we performed an analysis in which continuous outcome variables were replaced with quintiles, with the lowest quintile as a reference. The *E*-value (Ding and VanderWeele, 2016; VanderWeele and Ding, 2017), defined as the minimum association required to completely cancel out the observed association, was calculated to evaluate unmeasured confounding. In this study, the value was calculated using the risk ratio (RR) derived from the maximum and minimum adjusted prevalence in the range of the 0.25th to 99.75th percentile.

#### Additional analysis

Multicollinearity was assessed using generalised variance inflation factors.

## Results

In total, 48 065 male–female pairs were analysed; 63.4% of the male partners were younger than 35 years of age, 69.0% had a body mass index of 18.5–25.0 and 34.2% had at least 16 years education. Characteristics of the male partners are presented in Table 1. Compared with pregnant women whose partners participated (*n* = 48 065), those whose partners did not (*n* = 49 348) tended to be unmarried (Cramer's *V* = 0.079), to have one or more children (Cramer's *V* = 0.077), to experience IPV (Cramer's *V* = 0.047), to smoke (Cramer's *V* = 0.042) and to not be employed (Cramer's *V* = 0.040).

Energy-adjusted omega-3 PUFA values for the 0.25th, 25th, 50th, 75th and 99.75th percentiles were 0.14, 1.39, 1.81, 2.27 and 5.10 g/day, respectively. The distribution of intake became sparser at higher levels. The prevalence of physical violence was 1.13% (*n* = 545), and that of emotional abuse was 12.46% (*n* = 5991). The prevalence of each IPV pattern was as follows: both physical violence and emotional abuse, 0.94% (*n* = 451); physical violence only, 0.19% (*n* = 94); emotional abuse only, 11.53 (*n* = 5540); and none, 87.34% (*n* = 41 980), respectively.

**Fig. 2.**
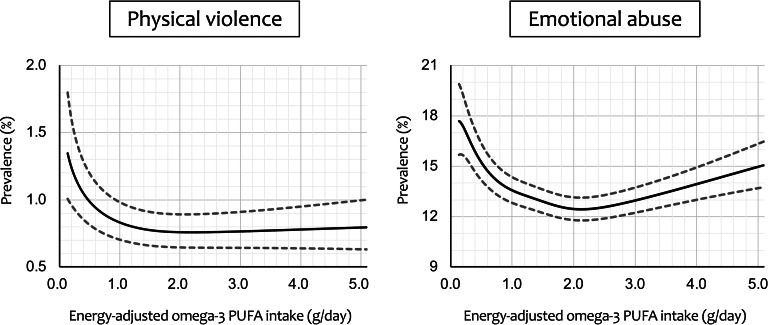
Regression curve with energy-adjusted omega-3 polyunsaturated fatty acid (PUFA) intake on the *x*-axis and adjusted^a^ prevalence of intimate partner violence on the *y*-axis. Dashed lines represent the ± standard error of the mean. ^a^Adjusted for male partner's age, body mass index, highest education level, annual household income, permanent full-time work, alcohol intake, smoking status, marital status, number of children, history of major psychiatric disease and severe psychological distress, with the 15 regional centres set as a random effect.

Figure 2 shows regression curves of adjusted prevalence ± SEM for the cases of physical violence and emotional abuse *v.* energy-adjusted omega-3 PUFA intake, calculated by generalised additive mixed-model analysis. A sharply decreasing regression curve with increasing omega-3 PUFA intake was plotted for physical violence. The prevalence started at 1.35% at the lowest intake level and decreased to a minimum value of 0.76% at 2.20 g/day (71.7th percentile). However, the prevalence largely remained flat primarily from there onward, with the upper limit of the error range not reaching the initial lower limit of the error range (1.01%) until intake exceeded 5.21 g/day (99.77th percentile). For emotional abuse, on the other hand, there was a more typical but distorted U- or V-shaped regression curve that initially decreased sharply and then rose slightly after reaching a minimum. The prevalence declined from 17.69% initially, reached a minimum value of 12.44% at 2.13 g/day (68.3th percentile) and then rose slightly. The lower limit of the error range reached the minimum upper limit (13.13%) at 4.17 g/day (99.11th percentile), and then the upper limit of the error range reached the maximum lower limit value (15.71%) at 4.56 g/day (99.48th percentile).

The analysis replacing continuous omega-3 PUFA intake with quintiles revealed that adjusted ORs with 95% CIs for physical violence, with the third quintile as the reference category, were as follows: first quintile, 1.44 (1.10–1.90); second quintile, 1.28 (0.96–1.71); fourth quintile, 1.29 (0.96–1.73); and fifth quintile, 1.28 (0.95–1.73). Adjusted ORs (95% CIs) for emotional abuse, with the fourth quintile as the reference category, were as follows: first quintile, 1.15 (1.06–1.26); second quintile, 1.12 (1.02–1.22); third quintile, 1.04 (0.95–1.14); and fifth quintile, 1.10 (1.01–1.20). Overall, these results were consistent with those derived from the main analysis. The *E*-values for physical violence (RR = 1.78) and emotional abuse (RR = 1.42) were 2.95 and 2.20, respectively. These values suggest that relatively strong unmeasured potential confounders would be necessary to cancel out the observed association.

All generalised variance inflation factors were less than 1.32, indicating the absence of multicollinearity among all covariates.

## Discussion

Our analysis obtained three main findings. First, except for extremely high intake, a higher intake of omega-3 PUFAs by male partners was associated with lower risk of IPV perpetration in general. Second, the association was observed for both physical violence and emotional abuse. Third, the overall association formed a distorted U-shaped curve with a relative sharp decrease on the left and a relatively slow increase on the right.

The first result is consistent with the evidence that omega-3 PUFA intake suppresses aggressive and violent behaviours, which was derived from a meta-analysis (Gajos and Beaver, 2016) that identified 73 effect sizes from 40 studies. These findings were obtained via DHA and/or EPA supplementation or measurement. Previous studies have pointed out that the effects of omega-3 PUFAs may be augmented in stressful and/or vulnerable situations (Appleton *et al*., 2008; Hamazaki and Hamazaki, 2008). Even though conception is generally regarded as a happy event, it can be stressful for the male partners (Holmes and Rahe, 1967). Our results suggest that the favourable effect of omega-3 PUFAs is applicable to IPV perpetration, which is a new finding from this work.

The second finding is also consistent with the existing evidence (Gajos and Beaver, 2016). Because the evidence was not limited to specific kinds of aggression and/or hostility, our results regarding physical violence and emotional abuse can also be placed into the same framework and interpreted together with the existing evidence. However, it should be noted that our IPV measures were not the standard semi-structured interview used in the WHO Multi-country Study on Women's Health and Domestic Violence against Women (Garcia-Moreno *et al*., 2006), did not include items regarding sexual violence and controlling behaviour, and consisted of only one question each for physical violence and emotional abuse. In this sense, our data may not give a complete picture of IPV. Nonetheless, physical violence and emotional abuse items show high inter-item correlations (Fulu *et al*., 2013), so it is hard to regard our items as measuring something completely different. Our items do in essence address a certain kind of aggression and/or hostility. Thus, although our results cannot be compared with the prevalence of IPV from other studies, this does not necessarily preclude the present association between male intake of omega-3 PUFAs and IPV perpetration. Further work using standard outcome measures for IPV perpetration is necessary.

The third finding is consistent with previous results concerning the association between maternal omega-3 intake and postpartum depression (Hamazaki *et al*., 2018, 2020). Such a U-shape association was also observed in those studies, whether significant or not, even though the exposure variable was the intake of omega-3 PUFAs by pregnant women rather than by male partners. While the underlying mechanism explaining these trends of an initial decrease and a subsequent increase is not yet known, a common explanation is that both deficiency and excess are unfavourable, as with many nutrients or drugs. Interestingly, there is a metabolic route for omega-3 that fits this explanation well (Mischoulon *et al*., 2009). Besides the underlying mechanism, the lowest risk was found around the 70th percentile in this study, which would be a relatively high percentile for many populations. This is because this study was conducted in Japan, and the Japanese population is known to eat large amounts of fish. For example, in 2010, when the JECS began, apparent seafood consumption in Japan ranked second among G20 countries at 52.7 kg/year, which is by far the highest among G20 countries, compared to 21.4 kg in the USA, 19.7 kg in the UK and so on (Food and Agriculture Organization of the United Nations, 2022). Considering that the risk showed the sharpest decrease at lower levels of omega-3 PUFA intake, where the distribution of intake was densest, even a small increase in fish consumption regardless of the exact amount might lead to an overall reduction in IPV, especially for men who do not eat fish. However, the same benefit of simply encouraging any increase in fish consumption would not be expected in countries with high fish consumption, such as Iceland, where apparent seafood consumption is reported to be 89.9 kg/year (Food and Agriculture Organization of the United Nations, 2022).

Many risk factors for IPV have been identified (Krug *et al*., 2002; Abramsky *et al*., 2011; World Health Organization and Pan American Health Organization, 2012; Fulu *et al*., 2013), but as mentioned above, these include young age, fewer years of education, poverty and substance abuse. As such, it would be difficult to devise interventions targeting these factors. In contrast, it is easier to encourage people who are lacking in omega-3 PUFAs to eat more omega-3-rich fish with a high fat content, such as saury and pilchard. Conveniently, blue-backed fishes are small and are not at the top of the food chain, so biological concentrations of toxic chemicals such as mercury and/or polychlorinated biphenyl are low. Given that a pregnant woman and her male partner are likely to share meals and have similar diets, it would be safer to avoid large fish or mammals rich in omega-3 PUFAs, such as fatty tuna and whale skin, as they tend to contain relatively high levels of toxic substances. Other recommendations on fish consumption can be found elsewhere in the literature (Mozaffarian and Rimm, 2006; Coletta *et al*., 2010) and in local fish consumption guidelines for pregnant women. Considering our results together with the many other health benefits of eating fish (Mozaffarian and Rimm, 2006; Akintoye *et al*., 2018), we recommend that fish intake be increased.

This study has several strengths. First, our sample size was large, including over 48 000 male–female pairs, which resulted in the successful detection of differences in physical abuse with a low prevalence of about 1.13%. Second, the participants were enrolled from multiple regions throughout Japan from 2011 to 2014 and are therefore possibly reflective of the entire nation. Third, the missing value rate was relatively low (⩽2% for most variables); this indicates small selection bias, apart from the non-participation of about half of male partners.

This study has several limitations. First, because we recruited all participants in a face-to-face manner, we were able to reach only those partners who attended visits with pregnant women. Although the reason for non-participation included availability, our participants nonetheless comprised male partners who desired to be involved in the pregnancy, suggesting selection bias. Second, we did not use the standard semi-structured interview and our prevalence of IPV might therefore differ from the true picture. In addition, we used very simple items asking only one question each for physical violence and emotional abuse, and the validity of these items may be questionable, although our items do address a certain kind of aggression and/or hostility. Third, we did not measure sexual violence or controlling behaviour and it is therefore unclear whether our findings could be generalised to these kinds of IPV. Fourth, we cannot rule out the possibility that IPV was underreported by the women who responded to the survey, although the answer refusal rate was far lower for items asking about IPV than for those asking about income, suggesting that such underreporting is not a major concern. Fifth, we used a validated version of the food frequency questionnaire, but the validation was not specific to these participants. In addition, the food frequency questionnaire method is generally less accurate than the food weighing method. Thus, the measured value of omega-3 PUFA intake may include variations or error. Finally, we did not measure adverse childhood experiences and/or child abuse experiences of the male partners during their own childhood. Although the effect of such experiences on omega-3 PUFA intake is unclear and does not seem to fulfil the necessary prerequisites for possible confounders, it would be better to measure these variables.

## Conclusions

The results of this study indicate an association between higher male intake of omega-3 PUFAs and lower risk of IPV perpetration, except for extremely high intake. Male consumption of omega-3-rich fish might be helpful in preventing IPV perpetration.

## Data

The data used to derive our conclusions are currently unsuitable for public deposition because of ethical restrictions and the specific legal framework in Japan. Furthermore, the Ethical Guidelines for Epidemiological Research enforced by the Japanese Ministry of Education, Culture, Sports, Science, and Technology and the Ministry of Health, Labour, and Welfare restrict the open sharing of epidemiological data. All inquiries about access to data should be sent to: jecs-en@nies.go.jp. The person responsible for handling enquiries at this e-mail address is Dr Shoji F. Nakayama, JECS Programme Office, National Institute for Environmental Studies.

## Appendix

Members of the JECS Group as of 2021: Michihiro Kamijima (Principal Investigator, Nagoya City University, Nagoya, Japan), Shin Yamazaki (National Institute for Environmental Studies, Tsukuba, Japan), Yukihiro Ohya (National Center for Child Health and Development, Tokyo, Japan), Reiko Kishi (Hokkaido University, Sapporo, Japan), Nobuo Yaegashi (Tohoku University, Sendai, Japan), Koichi Hashimoto (Fukushima Medical University, Fukushima, Japan), Chisato Mori (Chiba University, Chiba, Japan), Shuichi Ito (Yokohama City University, Yokohama, Japan), Zentaro Yamagata (University of Yamanashi, Chuo, Japan), Hidekuni Inadera (University of Toyama, Toyama, Japan), Takeo Nakayama (Kyoto University, Kyoto, Japan), Hiroyasu Iso (Osaka University, Suita, Japan), Masayuki Shima (Hyogo College of Medicine, Nishinomiya, Japan), Hiroshige Nakamura (Tottori University, Yonago, Japan), Narufumi Suganuma (Kochi University, Nankoku, Japan), Koichi Kusuhara (University of Occupational and Environmental Health, Kitakyushu, Japan), and Takahiko Katoh (Kumamoto University, Kumamoto, Japan).
